# Lithium cobalt(II) pyrophosphate, Li_1.86_CoP_2_O_7_, from synchrotron X-ray powder data

**DOI:** 10.1107/S1600536811038451

**Published:** 2011-09-30

**Authors:** Hui Zhou, Shailesh Upreti, Natasha A. Chernova, M. Stanley Whittingham

**Affiliations:** a Chemistry and Materials, SUNY Binghamton, Binghamton, NY, USA

## Abstract

Structure refinement of high-resolution X-ray powder diffraction data of the title compound gave the composition Li_1.865_CoP_2_O_7_, which is also verified by the ICP measurement. Two Co sites exist in the structure: one is a CoO_5_ square pyramid and the other is a CoO_6_ octa­hedron. They share edges and are further inter­connected through P_2_O_7_ groups, forming a three-dimensional framework, which exhibits different kinds of inter­secting tunnels containing Li cations and could be of great inter­est in Li ion battery chemistry. The structure also exhibits cation disorder with 13.5% Co residing at the lithium (Li1) site. Co seems to have an average oxidation state of 2.135, as obtained from the strutural stochiometry that closely supports the magnetic susceptibility findings.

## Related literature

For related structures, see: Adam *et al.* (2008 ▶); Nishimura *et al.* (2010 ▶); Zhou *et al.* (2011 ▶). For related materials with Na^+^ and K^+^ cations, see: Erragh *et al.* (1991 ▶); Sanz *et al.* (1999 ▶); Beaury *et al.* (2004 ▶); Gopalakrishna *et al.* (2005 ▶); Bih *et al.* (2006 ▶); Guesmi *et al.* (2007 ▶). For related structural frameworks, see: Beaury *et al.* (2004 ▶); Fagginani & Calvo (1976) ▶; Sandström *et al.* (2003 ▶); Etheredge & Hwu (1995 ▶); El Maadi *et al.* (1995 ▶); Huang & Hwa (1998 ▶); Sanz *et al.* (1999 ▶); Erragh *et al.* (1998 ▶). Pseudovoigt profile coefficients as parameterized in Thompson *et al.* (1987 ▶) and Finger *et al.* (1994 ▶).

## Experimental

### 

#### Crystal data

CoLi_1.865_O_7_P_2_
*M* *_r_* = 245.82
Monoclinic, 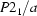

*a* = 9.76453 (4) Å
*b* = 9.69622 (4) Å
*c* = 10.95952 (4) Å
β = 101.7664 (2)°
*V* = 1015.83 (1) Å^3^
*Z* = 8
Synchrotron radiation, λ = 0.413988 Å
μ = 0.89 mm^−1^
*T* = 297 K
irregular shape, 15 × 13 mm

#### Data collection

Advanced Photon Source diffractometer
Specimen mounting: kapton capillary
Data collection mode: transmission
Scan method: continuous

#### Refinement

*R*_p_ = 0.057
*R*_wp_ = 0.080
*R*_exp_ = 0.049
*R*(*F* ^2^) = 0.04534
χ^2^ = 2.624
24500 data points
269 parameters

### 

Data collection: Advance Photon Source Argonne National Laboratory; cell refinement: *GSAS* (Larson & Von Dreele, 2000 ▶); data reduction: *Powder4* (Dragoe, 2001 ▶); program(s) used to solve structure: *GSAS*; program(s) used to refine structure: *GSAS*; molecular graphics: *CrystalMaker* (Palmer, 2005 ▶); software used to prepare material for publication: *publCIF* (Westrip, 2010 ▶).

## Supplementary Material

Crystal structure: contains datablock(s) I. DOI: 10.1107/S1600536811038451/br2175sup1.cif

Rietveld powder data: contains datablock(s) I. DOI: 10.1107/S1600536811038451/br2175Isup2.rtv

Additional supplementary materials:  crystallographic information; 3D view; checkCIF report

## Figures and Tables

**Fig. 1.**
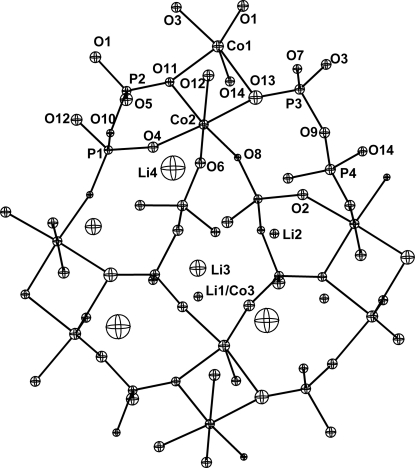
Thermal ellipsoid view of Li1.86CoP2O7 framework, having edge shared CoO5 and CoO6 interconnected through P2O7 moities.

**Fig. 2.**
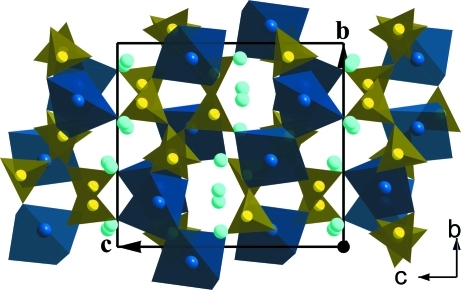
Polyhedral view of unit cell packing showing tunneled structures containing Li ions, viewed along [100].

**Fig. 3.**
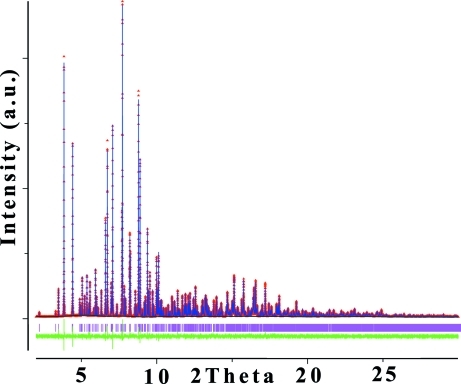
X-ray Rietveld refinement profiles for Li1.86CoP2O7, data recorded at room temperature. Triangles mark the experimental points (red), solid line is the calculated profile (blue) and bottom trace shows the difference curve (green).

## supplementary crystallographic information

### Comment

A_2_MP_2_O_7_ is a large family, in which various frameworks are encountered consisting of MO_6_ octahedra (Fagginani *et al.*, 1976; Sandström *et.al.*, 2003) and MO_4_ polyhedra (Etheredge *et al.*, 1995; Erragh *et al.*, 1998; Sanz *et al.*, 1999) interconnected through P_2_O_7_ groups (Fig. 1). Dimeric *M*_2_O_10_ units, built up of two edge-sharing MO_6_ octahedra, are only observed for some A_2_MP_2_O_7_ pyrophosphates (El Maadi *et al.* 1995; Huang *et al.* 1998) and dimeric *M*_2_O_11_ units(corner-shared MO_6_) seem to be much more rare, just observed for Na_2_CoP_2_O_7_ (Erragh *et al.* 1991). Two forms of structures were found for Na_2_CoP_2_O_7_ by Erragh *et al.* 1991: one is triclinic and another one is orthorhombic. The tetragonal structure of Na_2_CoP_2_O_7_ was reported by Sanz *et al.* 1999 and they found that the tetragonal form could be a derivative of the orthorhombic form, with a higher point symmetry for the former. In addition, the tetragonal structured Na_2_CoP_2_O_7_ was described by Guesmi *et al.* 2007. To our knowledge, the A_2_CoP_2_O_7_ with Li as cation has never been reported.

Here, we report a new Li containing solid with a three-dimensional framework (Fig. 2) crystallizing in the monoclinic space group *P*2_1_/*a*. Its structure is similar to the recently reported Li_2_MnP_2_O_7_ (Adam *et al.* 2008), a new member of the A_2_MP_2_O_7_ family: original *M*_2_O_9_ units, built up of one MO_5_ trigonal bipyramid sharing one edge with one MO_6_ octahedron, sharing corners with P_2_O_7_ pyrophosphate groups to form undulating (*M*_4_P_8_O_32_)_∞_ layers. A 3-D framework results from the interconnection between metal oxide and pyrophosphate groups, and the lithium cations are located in the tunnels thus formed (Fig 2). The structure of the related Fe-compound has been studied by us (Zhou *et al.* 2011) and Nishimura *et al.* (2010), as well as the electrochemical properties, which showed that it is a good candidate for the cathode material of lithium-ion batteries. The title compound also has the potential to work as the cathode material for lithium-ion batteries. We present here its crystal structure, as determined and refined from synchrotron powder X-ray diffraction data (Fig. 3).

### Experimental

The powder sample was synthesized through a "wet" method based on mixing stoichiometric aqueous solutions of the precursors followed by thermal treatments. The general procedure involves the mixing of soluble precursors in distilled water followed by a slow evaporation through continuous stirring to dryness before annealing the resultant solids. The precursors for the synthesis were Li(CH_3_COO),Co(CH_3_COO)_2_. 4H_2_O,and NH_4_H_2_PO_4_, which were dissolved in 100 ml distilled water in a molar ratio of 2:1:2 (1.32 g, 2.49 g and 2.3 g respectively) to give a 0.02 molar lithium solution. The self-adjusted pH of all the solutions were found to be around 4.5. The solution was stirred and evaporated on a hot-plate in the hood followed by vacuum oven drying overnight at 363 K. The resulting solid was preheated in a H2/He (8.5°/91.5° by volume) atmosphere at 673 K for 4h to decompose the precursors followed by reheating under the same atmosphere up to 873 K for 16h with intermittent grinding to obtain the pink colored powder as final product. The sample was also analyzed with a Perkin-Elmer ICP-OES Optima 7000 DV for the elemental content. The average result of 3 analyses showed that the ratio of Li: Co: P is 1.85: 0.996:2. In addition, the SQUID magnetic study on the sample using a Quantum Design MPMS XL SQUID magnetometer showed that the effective magnetic moment of it is 5.23*m*_B_which is typical divalent Co.

### Refinement

During structural refinement, occupancy factor for Li1 and Co3 were refined using constrains for atomic coordinate, atomic displacement parameter, and keeping the sum of occupancy facter equals to unity, which later were fixed to their close refined values as 0.73 and 0.27 respectively. Occupancy for Co1 was also observed to be deficient and fixed to it's closely refined value of 0.739 to 0.73, in final refinement cycles.

### Crystal data

**Table:**

CoLi_1.865_O_7_P_2_	*F*(000) = 948.7
*M**_r_* = 245.82	*D*_x_ = 3.214 Mg m^−^^3^
Monoclinic, *P*2_1_/*a*	Melting point: 1023 K
Hall symbol: -P 2yab	Synchrotron radiation, λ = 0.413988 Å
*a* = 9.76453 (4) Å	µ = 0.89 mm^−^^1^
*b* = 9.69622 (4) Å	*T* = 297 K
*c* = 10.95952 (4) Å	Particle morphology: block
β = 101.7664 (2)°	pink
*V* = 1015.83 (1) Å^3^	irregular, 15 × 13 mm
*Z* = 8	Specimen preparation: Prepared at 873 K and 101.325 kPa

### Data collection

**Table:**

Advanced Photon Source diffractometer	Specimen mounting: kapton capillary
Radiation source: Synchrotron	Data collection mode: transmission
Si	Scan method: continuous

### Refinement

**Table:**

Least-squares matrix: full	Excluded region(s): Reflections exceeding 2-theta 30 were omitted for the ease of refinement.
*R*_p_ = 0.057	Profile function: CW Profile function number 3 with 19 terms Pseudovoigt profile coefficients as parameterized in Thompson *et al.*, (1987) and Finger *et al.* (1994).#1(GU) = 6.454 #2(GV) = -0.998 #3(GW) = 0.075 #4(GP) = 0.000 #5(LX) = 0.327 #6(LY) = 0.000 #7(S/L) = 0.0011 #8(H/L) = 0.0014 #9(trns) = 0.00 #10(shft)= 0.0000 #11(stec)= 0.00 #12(ptec)= 0.00 #13(sfec)= 0.00 #14(L11) = 0.067 #15(L22) = 0.070 #16(L33) = 0.058 #17(L12) = 0.010 #18(L13) = 0.010 #19(L23) = -0.004 Peak tails are ignored where the intensity is below 0.0010 times the peak Aniso. broadening axis 0.0 0.0 1.0
*R*_wp_ = 0.080	269 parameters
*R*_exp_ = 0.049	0 restraints
*R*(*F*^2^) = 0.04534	(Δ/σ)_max_ = 0.03
χ^2^ = 2.624	Background function: GSAS Background function number 1 with 36 terms. Shifted Chebyshev function of 1st kind 1: 165.338 2: -31.4210 3: -8.19118 4: 6.28432 5: -10.8524 6: 14.3842 7: -8.22810 8: -0.949190 9: 13.3092 10: -14.8044 11: 4.75285 12: -1.09442 13: -0.739293 14: 5.47743 15: -2.87265 16: 1.05489 17: -3.22764 18: 1.21714 19: 0.537209 20: -3.25962 21: 2.18736 22: -1.12437 23: -2.15219 24: 3.41374 25: -3.88852 26: 1.14500 27: 3.06741 28: -3.05038 29: 0.529274 30: 0.298855 31: -4.06399 32: 1.50867 33: 1.15056 34: -2.20673 35: 0.550944 36: 5.540140E-02
24500 data points	

### Special details

**Table:**

Experimental. Data was collected with powder sample packed in Kapton capillary

### Fractional atomic coordinates and isotropic or equivalent isotropic displacement parameters (Å^2^)

**Table:**

	*x*	*y*	*z*	*U*_iso_*/*U*_eq_	Occ. (<1)
Co1	0.25281 (15)	0.71550 (15)	0.18010 (14)	0.0137 (4)*	0.73
Co2	0.30165 (11)	0.43043 (11)	0.32719 (10)	0.0095 (3)*	
P1	0.3790 (2)	0.6536 (2)	0.5771 (2)	0.0078 (5)*	
P2	0.0613 (2)	0.9265 (2)	0.24116 (18)	0.0089 (5)*	
P3	0.0210 (2)	0.4551 (2)	0.75861 (19)	0.0103 (5)*	
P4	0.6144 (2)	0.7956 (2)	−0.1113 (2)	0.0117 (6)*	
O1	0.1615 (5)	0.8210 (5)	0.3113 (5)	0.0130 (14)*	
O2	0.4709 (5)	0.7806 (5)	−0.0792 (4)	0.0106 (12)*	
O3	0.3911 (5)	0.8599 (4)	0.1472 (5)	0.0107 (13)*	
O4	0.0302 (5)	0.8437 (5)	0.5592 (4)	0.0116 (14)*	
O5	0.4330 (5)	0.4292 (5)	−0.1033 (4)	0.0168 (13)*	
O6	0.1790 (5)	0.8356 (5)	−0.1511 (4)	0.0115 (13)*	
O7	0.0189 (5)	0.4120 (4)	0.6224 (4)	0.0087 (13)*	
O8	0.1866 (5)	0.2976 (4)	0.4146 (4)	0.0047 (12)*	
O9	0.1013 (5)	0.6017 (4)	0.7747 (4)	0.0116 (13)*	
O10	0.3827 (5)	0.5734 (5)	0.7087 (4)	0.0061 (12)*	
O11	0.4152 (5)	0.5900 (4)	0.2661 (4)	0.0085 (13)*	
O12	0.2775 (5)	0.5646 (5)	0.4803 (4)	0.0134 (13)*	
O13	0.3713 (5)	1.0314 (5)	−0.2124 (5)	0.0205 (15)*	
O14	0.2204 (5)	0.6299 (5)	−0.0018 (4)	0.0094 (13)*	
Li1	1.3433 (3)	0.9255 (3)	−0.0397 (3)	0.0087 (7)*	0.73
Li2	0.0807 (17)	0.1083 (15)	0.0261 (15)	0.009 (4)*	
Li3	0.6728 (15)	0.0711 (14)	0.5465 (14)	0.029 (4)*	
Li4	0.400 (2)	0.246 (2)	0.5725 (17)	0.065 (7)*	
Co3	1.3433 (3)	0.9255 (3)	−0.0397 (3)	0.0087 (7)*	0.27

### Geometric parameters (Å, °)

**Table:**

Co1—O1	2.105 (5)	P2—O5^v^	1.524 (4)
Co1—O3	2.028 (4)	P2—O10^iv^	1.584 (5)
Co1—O11	2.067 (4)	P2—O11^vi^	1.516 (5)
Co1—O13^i^	2.226 (5)	P3—O3^ii^	1.514 (5)
Co1—O14	2.123 (5)	P3—O7	1.546 (5)
Co2—O4^ii^	2.030 (5)	P3—O9	1.616 (4)
Co2—O6^i^	2.180 (5)	P3—O13^vii^	1.563 (5)
Co2—O8	2.068 (4)	P4—O2	1.519 (5)
Co2—O11	2.091 (4)	P4—O6^iii^	1.524 (4)
Co2—O12	2.173 (4)	P4—O9^viii^	1.582 (5)
Co2—O13^i^	2.128 (5)	P4—O14^iii^	1.589 (5)
P1—O4^iii^	1.529 (5)	Co3—O2^ix^	1.983 (5)
P1—O8^iv^	1.547 (4)	Co3—O3^ix^	2.104 (6)
P1—O10	1.633 (5)	Co3—O6^ix^	2.006 (6)
P1—O12	1.556 (4)	Co3—O13^ix^	2.220 (5)
P2—O1	1.512 (5)	Co3—O14^x^	2.154 (5)
			
O1—Co1—O3	100.14 (19)	O8—Co2—O11	169.24 (17)
O1—Co1—O11	111.53 (19)	O8—Co2—O13^i^	96.85 (19)
O1—Co1—O14	145.94 (21)	O11—Co2—O13^i^	83.05 (18)
O3—Co1—O11	90.63 (18)	O8^iv^—P1—O10	108.18 (27)
O3—Co1—O14	94.60 (20)	O8^iv^—P1—O12	109.11 (29)
O11—Co1—O14	98.73 (18)	O10—P1—O12	103.57 (26)
O4^ii^—Co2—O8	84.60 (18)	O1—P2—O5^v^	111.42 (29)
O4^ii^—Co2—O11	95.03 (18)	O1—P2—O10^iv^	106.90 (27)
O4^ii^—Co2—O13^i^	177.02 (20)	O5^v^—P2—O10^iv^	104.39 (29)

Symmetry codes:  (i) −*x*+1/2, *y*−1/2, −*z*; (ii) −*x*+1/2, *y*−1/2, −*z*+1; (iii) *x*+1/2, −*y*+3/2, *z*; (iv) −*x*+1/2, *y*+1/2, −*z*+1; (v) −*x*+1/2, *y*+1/2, −*z*; (vi) *x*−1/2, −*y*+3/2, *z*; (vii) *x*−1/2, −*y*+3/2, *z*+1; (viii) *x*+1/2, −*y*+3/2, *z*−1; (ix) *x*+1, *y*, *z*; (x) −*x*+3/2, *y*+1/2, −*z*.
